# The Influence of Bleaching Intensity and Laser Activation on the Durability of Selected Aesthetic Composites—An In Vitro Study

**DOI:** 10.3390/jfb16060193

**Published:** 2025-05-23

**Authors:** Żaneta Anna Mierzejewska, Kamila Łukaszuk, Bartłomiej Rusztyn, Kacper Maliszewski

**Affiliations:** 1 Institute of Biomedical Engineering, Faculty of Mechanical Department, Bialystok University of Technology, 15-351 Bialystok, Poland; k.maliszewski231@gmail.com; 2 Department of Maxillofacial and Plastic Surgery, Medical University of Bialystok, 15-089 Białystok, Poland; bartlomiej.rusztyn@umb.edu.pl

**Keywords:** composite resins, bleaching, surface roughness, microhardness

## Abstract

The aim of this study was to evaluate the effect of different bleaching protocols, including laser-assisted techniques, on the microhardness, surface roughness, and tribological resistance of selected light-cured composites. Three Estelite Universal Flow composites with different flow properties and G-aenial Universal Flo composites were tested. Each group underwent bleaching procedures using Opalescence agents at 10%, 16%, and 40% concentrations, with and without laser activation. Surface microhardness was assessed using the Vickers method, roughness was measured with 3D confocal laser microscopy, and friction coefficients and wear patterns were evaluated using tribological testing. All bleaching protocols resulted in reduced microhardness and increased surface roughness. The most significant changes were observed after treatment with 40% hydrogen peroxide. Laser application, particularly at 16% concentration of carbamide peroxide, helped to partially mitigate these effects in some materials. Bleaching procedures, especially those involving high peroxide concentrations, significantly deteriorated the surface properties of dental composites, which may have clinical implications for the patients.

## 1. Introduction

Teeth whitening has become one of the most popular aesthetic treatments in dentistry. Both at home and in the office, patients are increasingly using whitening products. Although these substances are effective in removing discoloration and restoring the natural whiteness of teeth, their impact on the hard structures of teeth and dental fillings is of interest to scientists and clinicians [1,2,3]. The main ingredient of whitening agents is hydrogen peroxide or its derivative, carbamide peroxide, which decomposes into hydrogen peroxide and urea. H_2_O_2_ works by releasing active oxygen, which breaks down the bonds in the molecules of pigments responsible for enamel discoloration. This process takes place at the chemical level, without significant interference with the enamel structure, but the intensity of the treatment and the concentration of whitening substances may cause certain changes in dental fillings [4,5,6,7].

Dental composites are a key material in modern conservative dentistry, combining aesthetics with high mechanical strength. Their structure is based on two main components: a polymer matrix and fillers, whose properties largely determine the mechanical parameters and durability of the filling [1]. The matrix, usually composed of dimethacrylate resins such as bis-GMA, UDMA, or TEGDMA, acts as a binder, which after polymerization creates a rigid polymer network that is at the same time susceptible to mechanical loads [8,9]. Fillers, which are inorganic particles, are the second key component, the mass fraction of which can reach up to 70–85% of the entire material. They affect not only the hardness, modulus of elasticity, and abrasion resistance, but also the polymerization shrinkage and aesthetics of the composite. Modern fillers differ in composition (silica, barium glass, nanoparticles) and size—from nanometers to several micrometers. The size, shape, and distribution of filler particles affect light transmittance, optical properties, and the degree of matrix conversion [10]. Optimization of the matrix to filler ratio, as well as the appropriate selection of their physicochemical properties, is crucial to obtaining a material with the desired clinical parameters, such as high mechanical strength, low polymerization shrinkage, chemical durability, and appropriate aesthetics [11,12]. In practice, this means not only choosing the right size, shape, and percentage of filler, but also modifying the matrix itself. Examples include polymerization inhibitors and stabilizers such as hydroquinone (HQ) or butylated hydroxytoluene (BHT) which prevent premature polymerization of the resin during storage. Another key ingredient is photoinitiators, such as camphorquinone (CQ) or trimethylphosphinoxide (TPO), which are responsible for initiating the matrix cross-linking process under the influence of light [13]. High-molecular-weight monomers are also used to minimize polymerization shrinkage and improve dimensional stability. The use of silane molecules to modify the surface of fillers improves the bond strength between the matrix and the filler, which increases the bending resistance of the composite [14,15].

The selection of individual components of dental composites, as well as their percentage or mass share, is an extremely complex task. It requires taking into account many factors, such as mechanical, tribological, and thermal properties, as well as their mutual interactions and impact on the long-term stability of the material. The difficulty is that these properties are often interdependent, and modifying one component can affect others, sometimes in an unpredictable way. Hardness, modulus of elasticity, polymerization shrinkage, thermal conductivity, and wear resistance are interconnected, which means that optimizing the composition requires multi-faceted analysis. An interesting attempt at such a comprehensive analysis was presented by Yadav et al. (2024). In their study, they used the R-method (Ranking Method) to evaluate various composites filled with nanohydroxyapatite (nHAp). This work is distinguished by its holistic approach to the evaluation of materials, taking into account not only standard mechanical properties but also the behaviour of the material under friction and temperature changes, which is crucial in the context of real oral conditions [16].

Due to their complex structure, dental composites may exhibit varying resistance to hydrogen peroxide and carbamide peroxide. Under the influence of H_2_O_2_, the bonds between the organic resin and the filler are weakened, which leads to a gradual decrease in mechanical properties such as hardness, bending strength, or abrasion resistance [1,17]. This is due to the degradation process of the polymer matrix and the weakening of the bond between the fillers and the matrix. In addition, composites also contain organic compounds that can degrade under the influence of continuous exposure to chemicals contained in whitening pastes or other preparations. Over time, this can lead to microcracks, loss of surface smoothness, and a decrease in abrasion resistance [18,19,20,21].

The effect of H_2_O_2_ on fillings can increase the roughness of the composite surface, which promotes the accumulation of dental plaque and food dyes, and in the long term can lead to the formation of secondary stains [22]. Carbamide peroxide, due to its slower action, causes less intense mechanical changes, but long-term exposure can also lead to weakening of the material structure [23]. Reducing the microhardness of the dental composite surface is one of the main factors affecting its durability and long-term functioning. Microhardness is a key mechanical parameter that reflects the material’s resistance to local surface deformation under the influence of external forces. Reducing the microhardness of the composite surface directly affects its ability to cope with loads during everyday use, especially in chewing conditions [24,25,26]. Microhardness is closely correlated with other mechanical parameters of composites, such as material stiffness or elasticity. Material stiffness determines the ability of the filling to absorb mechanical forces during chewing. If the stiffness of the composite is too low, the material is unable to effectively distribute loads, which leads to excessive surface abrasion and, as a result, to a gradual loss of shape and durability of the filling. The elasticity (modulus of elasticity) of the composite plays an important role in preventing fracture [21,24,27]. Dental composites must be characterized by an appropriate ability to deform under load in order to absorb external forces, but at the same time, their return to the original shape must be controlled and not lead to fractures. Too low microhardness makes the composite more susceptible to microcracks, which over time can lead to greater structural damage, both superficial and deeper [28,29,30]. The correlation between microhardness and wear resistance indicates a relationship between surface properties and the overall strength of the material. Therefore, controlling the microhardness of the surface of composites is crucial to ensuring their long-term functionality and aesthetics [31,32].

In the context of the above considerations, the aim of this study was to assess in detail the surface condition of selected dental composites subjected to different bleaching protocols, used both in dental offices and at home. Therefore, the null hypothesis assumes that the use of active whitening agents, such as hydrogen peroxide or carbamide peroxide, will result in significant changes in the microhardness and surface roughness of dental composites.

## 2. Materials and Methods

The light-cured composites used in the study are as follows:

- Estelite Universal Flow with three different flowability levels.
- G-aenial Universal Flow (Table 1).

### 2.1. Sample Preparation

Sixty-five samples were made from each material—five for testing the unbleached surface (control samples) and ten for verifying the effect of each of the selected bleaching substances and procedures. In accordance with the ISO 20795-1:2008 standard, a duralumin matrix was used to produce the samples; this allowed us to obtain the desired shapes and dimensions. The composites were placed in the matrix using a ball feeder, and then the prepared form was placed between two glass plates covered with transparent, smooth polyester strips (TOR VM, Heidelberg, Germany). The material was then compressed with a glass plate. The samples were subjected to a load of 500 g for approximately 30 s to squeeze out excess composite and obtain flat surfaces. Each sample was polymerized according to the manufacturer’s recommendations, using a C01-C LED polymerization lamp (Premium Plus, United Kingdom) at a radiation intensity of 900 mW/cm^2^. The material was polymerized on both sides for 20 s each. The composite disks were polished with medium, fine, and extra-fine polishing disks (OptiDisc^®^, Kerr, Orange, MN, USA) and then with a polishing rubber point (Industriezone Neugut Liechtenstein). The samples were disk-shaped with a diameter of 8.0 ± 0.1 mm and a height of 2.5 ± 0.1 mm (after polishing). The samples were measured using a Mitutoyo IP65 digital micrometer (Mitutoyo Corporation, Kawasaki, Japan). After polishing, the samples were subjected for 2 min to ultrasonic cleaning with distilled water to remove any surface debris and conditioned to fully cross-link the composite in distilled water, then stored for 24 h at 37 °C [2].

### 2.2. Bleaching Procedure

To carry out the whitening process of composite samples, the Opalescence preparation was used, which consists of glycerin, water, carbamide peroxide, xylitol, carbomer, PEG-6, sodium hydroxide, EDTA, potassium nitrate, sodium fluoride, and active substances: carbamide peroxide and hydrogen peroxide. This preparation is available in three variants (concentrations):

- carbamide peroxide 10%, hydrogen peroxide (H_2_O_2_) 3.6%–Opalescence 10%;
- carbamide peroxide 16%, hydrogen peroxide (H_2_O_2_) 5.8%–Opalescence 16%;
- carbamide peroxide 40%, hydrogen peroxide (H_2_O_2_) 10.0%–Opalescence 40%.
- The bleaching procedure was carried out in accordance with the records presented in Table 2.

The whitening agent was applied to the meticulously finished and dried composite surface and incubated at 37 ± 2 °C for 6 h, replicating the conditions of the oral cavity in a moist and dim setting. The samples bleached in the Home bleaching system were washed with distilled water after the specified time and stored in a container with 0.9% NaCl saline at 37 °C between sessions. In parallel, the untreated control groups were maintained under identical conditions.

Opalescence 40% was applied in a dental office to exposed and dried teeth, after which composite samples were dried and coated with a layer of the active substance at room temperature (dental office temperature, May 2024, 23 °C) and maintained in such conditions. Samples bleached with additional use of laser pulses (diode laser, wavelength 810 nm, output 1.5 W, continuous mode) were irradiated for 90 s, and after this exposure, the active substance covered the sample surface for another 10 min. Between sessions, samples were rinsed with distilled water [1,33,34,35].

### 2.3. Microhardness Test

The iVick-450M microhardness tester (Dongguan Quality Control Technology Co., Ltd., Dongguan, China) was used for the tests. The measurement was performed using the Vickers method, using a diamond pyramid-shaped indenter with an angle of 136° between opposite walls. The indenter was pressed into the sample with a force of 0.98 N (load 0.1 kg) for 15 s [4]. Microhardness was determined based on the size of the indentation diagonals, calculated using the microhardness tester software. For each tested sample, 15 measurements were performed; the results were averaged, and the standard deviation was calculated.

### 2.4. Tribological Test

The UMT Tribolab tribometer (Bruker, Billerica, MA, USA) was used to determine the friction coefficients. A 6 mm diameter corundum ceramic ball with a roughness of Ra = 5.9 μm was used as the counter sample. The composite sample was mounted in a special holder attached to the device table. Friction occurred in a reciprocating motion, with an amplitude of 500 μm, a frequency of 1 Hz, a pressure force of 5 N, and an acquisition frequency of 10 Hz. The working temperature was 37 °C, and the working atmosphere was air. To simulate the physiological conditions in the oral cavity, artificial saliva (Xerostom Gel Saliva, Atos MM, Warsaw, Poland) was used as a lubricant in the study. The friction time was 60 min for each sample. The specific wear coefficients *K* were calculated with the following equation:

(1) $$K=\frac{W}{F\times L\;}\;\left[\frac{{mm}^{3}}{N\times mm}\right]$$

where *K* is the specific wear coefficient, *W* is the wear volume, *F* is the testing force, and *L* is the total sliding distance [36]. Three friction tests were performed for each sample, on both sides.

### 2.5. Roughness Test

To measure the surface roughness, a confocal 3D laser measuring microscope (OLYMPUS OLS 4000 3D, Tokyo, Japan) was used. The measurement methodology was developed in accordance with the PN-EN ISO 4288:2011 standard. On this basis, 5 elementary sections with a length of Le = 0.25 mm were determined for each sample. The stereometry of the surface layer was defined using the amplitude parameter Ra—the average value of all deviations from a straight line within the length of the elementary section [37].

### 2.6. Statistical Analysis

The statistical analysis was performed using Microsoft Excel and Statistica 13. The primary goal was to assess the effects of various bleaching treatments on the microstructural properties of composite materials, including microhardness (Table 3) and average surface roughness (Table 4). Paired *t*-tests were used to compare the mean microhardness and roughness values before and after bleaching for each of the four tested materials. One-way ANOVA was applied to determine whether the type of bleaching alone had a significant effect on the measured properties of each material. This test assessed the differences between multiple bleaching conditions (e.g., 10%, 16%, 40%, 10% + laser, 16% + laser, 40% + laser). The two-way ANOVA test provided insights into both the main effects and the interaction effects between these two factors, offering a more comprehensive understanding of the factors influencing microhardness and roughness. Regression analysis was used to quantify the strength and direction of the relationship between bleaching concentration and the changes in microhardness and roughness. The resulting R^2^ values indicated the proportion of the variance in the dependent variables that could be explained by the independent variable (bleaching concentration). All statistical tests were performed with a significance level of α = 0.05, with results considered statistically significant for *p* < 0.05.

## 3. Results

### 3.1. Microhardness Test

The microhardness results are presented as mean values with corresponding standard deviations, reflecting both the tendency and variability in the measurements, as shown in Table 3. G-aenial and Estelite Universal Flow High, as more viscous composite materials, demonstrate a noticeable decrease in microhardness as the bleaching concentration increases. For G-aenial, the control samples exhibit a mean microhardness of 47.29 (±0.71), which drops progressively to 33.99 (±0.87) with 40% bleaching. This represents a significant 28.1% reduction. Similarly, Estelite Universal Flow Hard shows a marked decline from a mean of 42.07 (±1.69) in control samples to 29.68 (±0.80) with 40% bleaching, reflecting a 29.5% reduction. These findings suggest the structural resilience of these composites, indicating substantial weakening of the material’s surface integrity under high-concentration bleaching.

Interestingly, the use of laser activation alongside bleaching generally mitigates hardness reduction for these two materials. For instance, G-aenial treated with 10% bleaching plus laser maintains a higher hardness of 46.70 (±0.44), close to its control value, while even 40% bleaching plus laser yields a much improved 38.50 (±0.21) compared to 33.99 (±0.87) without laser. A similar trend is observed for Estelite Universal Flow High, where the 10% bleaching plus laser condition results in a mean hardness of 38.40 (±0.39), substantially higher than that of the non-laser 10% condition (32.32 ± 0.75). This suggests that laser activation may play a protective role, potentially by reducing oxidative stress on the composite surface.

The medium and super low viscosity composites, Estelite Universal Flow Medium and Super Low, demonstrate more complex responses. The Estelite Universal Flow Medium generally retains its hardness better across different bleaching conditions, showing a slight reduction from 45.46 (±1.18) in control samples to 40.74 (±0.65) under the condition of bleaching 40% with laser. This roughly 10.4% drop is less severe than the declines observed for the more viscous materials. Notably, this material exhibits a slight increase in hardness under 40% bleaching alone (44.37 ± 1.58), possibly indicating some level of polymer cross-linking or surface hardening under intense oxidative conditions.

Estelite Universal Flow Super Low, known for its highly fluid consistency, reveals a unique trend where its hardness decreases significantly with 10% bleaching (46.52 ± 0.87) but stabilizes under higher concentrations. The 40% bleaching results in a hardness of 45.97 (±1.29), close to the value of 16% bleaching condition (45.01 ± 0.77) and notably higher than the value of the 10% bleached samples. This anomaly suggests that higher peroxide levels might promote a compensatory hardening effect, possibly through enhanced resin matrix polymerization or reduced plasticizing effects as volatile components are oxidized or removed.

The data in Table 3 show that all materials experienced significant reductions in microhardness after bleaching, as indicated by the paired *t*-tests (*p* < 0.05). The impact was strongest at higher bleaching concentrations, especially when combined with laser treatment, with G-aenial showing the largest decrease at 40% bleaching (t = 28.49, *p* < 0.001).The one-way ANOVA confirmed that bleaching type significantly affects microhardness for all materials (*p* < 0.001), while the two-way ANOVA showed significant effects for both bleaching type (F = 134.8) and material type (F = 78.4), with a strong interaction (F = 25.2, *p* < 0.001). Finally, the regression analysis revealed strong negative correlations between bleaching concentration and microhardness, with Estelite High (R^2^ = 0.95) being the most sensitive to bleaching and Estelite Super Low (R^2^ = 0.68) the least.

### 3.2. Tribological Test

Tribological tests were conducted on composite samples under seven experimental conditions, including a control (no bleaching), bleaching at 10%, 16%, and 40%, and the same concentrations combined with laser treatment. The coefficient of friction (COF) was measured over a 60 min period to assess the effects of these treatments on the surface properties of various dental composites.

**Figure 1:**
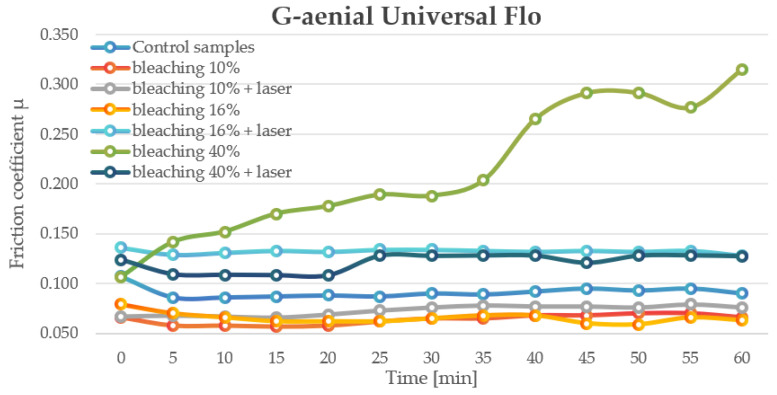
The change in motion resistance as a function of time: G-aenial Universal Flo.

G-aenial Universal Flo control samples exhibited a stable COF (~0.086–0.095). Bleaching at 10% significantly reduced the COF (0.057–0.07), maintaining the lowest values among all groups. However, adding laser treatment to 10% bleaching slightly increased COF (0.066–0.079), though it remained lower than the control. At 16% bleaching, COF decreased (0.059–0.068), but laser activation led to a notable increase (0.128–0.136), indicating potential surface damage. Bleaching at 40% sharply raised the COF (0.1065 to 0.315 over 60 min), while the addition of laser reduced this effect (0.1081–0.1284), suggesting partial mitigation of oxidative damage (Figure 1).

For Estelite Universal Flow High, the COF generally increased over time across all conditions. Control samples had a relatively stable COF (0.098–0.1190).

**Figure 2:**
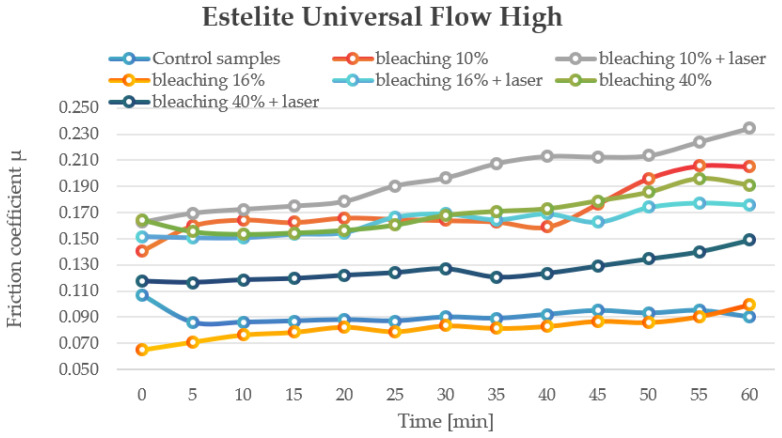
The change in motion resistance as a function of time: Estelite Universal Flow High.

Bleaching at 10% (0.14–0.205) and 16% (0.065–0.099) both increased the COF, with laser treatment further amplifying this effect, particularly at 10% (0.162–0.234). Bleaching at 40% (0.153–0.196) resulted in significant friction increases, though laser treatment reduced this range (0.116–0.149), indicating a complex interaction between concentration and laser activation (Figure 2).

**Figure 3:**
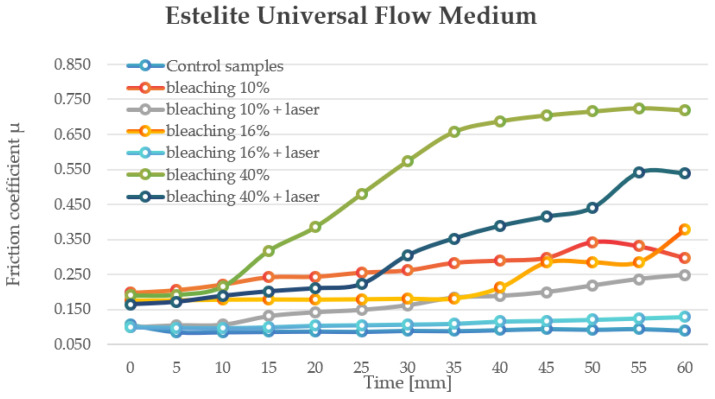
The change in motion resistance as a function of time: Estelite Universal Flow Medium.

Estelite Universal Flow Medium controls had the highest overall COF (0.485–0.716), with 10% (0.198–0.342) and 16% (0.175–0.378) bleaching significantly reducing the COF. The 16% + laser group demonstrated the lowest COF (0.097–0.129) and the most stable surface properties, while the 40% and 40% + laser groups had the highest COF variability (0.19–0.725 and 0.164–0.540, respectively), suggesting increased surface irregularity (Figure 3).

Estelite Universal Flow Super Low composites without bleaching exhibited an initial coefficient of friction (COF) of 0.289, which increased steadily over 60 min, reaching a peak of 0.714 at 55 min and stabilizing at 0.674. This pattern suggests progressive surface roughening, possibly due to micro-deformation or matrix restructuring.

**Figure 4:**
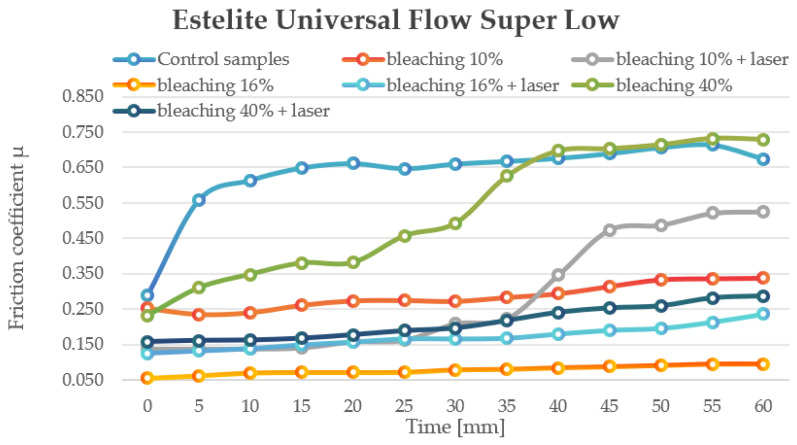
The change in motion resistance as a function of time: Estelite Universal Super Low.

Treatment with 10% bleaching reduced the initial COF to 0.252, though a gradual rise to 0.3375 over 60 min indicates that the friction-reducing effect is temporary. In contrast, 16% bleaching yielded the lowest COF among all tested groups, starting at 0.054 and only reaching 0.0945 after 60 min, highlighting its potential for long-term friction reduction in biomechanical applications. However, 40% bleaching had the opposite effect, with an initial COF of 0.2313 that sharply increased to 0.729, indicating significant surface damage, likely due to matrix erosion and microparticle exposure. Laser treatment significantly altered these trends. For 10% + laser, the initial COF was reduced to 0.138, but this benefit diminished over time, peaking at 0.525 after 60 min. In the 16% + laser group, the initial COF was 0.125, increasing to 0.236 over 60 min, indicating a more stable friction profile. The 40% + laser group demonstrated a notable reduction in the COF compared to non-laser samples, starting at 0.1575 and rising to 0.287, significantly lower than the 40% alone (0.729), suggesting that laser treatment can partially mitigate the negative effects of high bleach concentrations (Figure 4).

### 3.3. Microscopic Analysis 

The surface roughness (Ra) of dental composites is a critical factor in their long-term clinical performance. It affects wear resistance, aesthetics, and bacterial adhesion. The following analysis examines the effect of different bleaching protocols on the surface roughness of four composite materials: G-aenial Universal Flo and ESTELITE UNIVERSAL FLOW High, Medium, and Super Low. The results are presented in Table 4.

Control G-aenial Universal Flo samples exhibited the lowest roughness (Ra = 0.1982, ±0.0087), consistent with the absence of chemical or physical stress. Bleaching with 10% hydrogen peroxide significantly increased roughness to 0.278, the highest among all tested concentrations, suggesting that low-concentration agents may cause more aggressive surface etching. In contrast, 16% bleaching resulted in a lower Ra (0.2087), indicating that higher concentrations might produce more controlled surface oxidation, reducing microstructural damage. Bleaching at 40% showed a similar roughness (Ra = 0.2045) to bleaching at 16%, but with a higher standard deviation (0.0131), potentially reflecting increased instability at this concentration. Laser activation generally reduced roughness across all concentrations, with 40% + laser yielding the lowest Ra among the bleached samples (0.2106), possibly due to enhanced polymer cross-linking or reduced matrix disruption.

The Estelite Universal Flow High control samples had the lowest roughness (Ra = 0.11375). Bleaching at 10% (Ra = 0.1213) and 16% (Ra = 0.1216) resulted in modest roughness increases, reflecting moderate surface oxidation. However, the highest concentration (40%) produced the greatest roughness (Ra = 0.1253), suggesting more significant matrix degradation. Laser activation consistently reduced roughness, with 10% + laser (Ra = 0.1183), 16% + laser (Ra = 0.1198), and 40% + laser (Ra = 0.1167) showing progressive reductions, highlighting the potential stabilizing effect of laser on composite microstructure.

The control material Estelite Universal Flow Medium had the lowest roughness (Ra = 0.037), but roughness increased sharply with 10% bleaching (Ra = 0.0507, +37%), reflecting significant matrix disruption. Bleaching at 16% (Ra = 0.0498) produced similar roughness, while 40% bleaching had the highest Ra (0.069), indicating more extensive surface alteration. Interestingly, laser treatment at 40% reduced roughness (Ra = 0.059) compared to non-laser samples, suggesting that laser can mitigate high-concentration damage.

Estelite Universal Flow Super Low, with the lowest control roughness (Ra = 0.06), showed substantial roughness increases after 10% bleaching (Ra = 0.118), reflecting significant surface disruption. However, higher concentrations (16%, Ra = 0.081; 40%, Ra = 0.0836) produced comparatively lower roughness values, possibly due to more uniform chemical interaction. Laser treatment reduced roughness across all concentrations, with 10% + laser (Ra = 0.0634) and 16% + laser (Ra = 0.0642) approaching control values, indicating that laser may stabilize the matrix by reducing micropore formation.

The data indicate that all materials exhibited significant changes in surface roughness (Ra) after bleaching, as confirmed by the paired *t*-tests. The 10% bleaching treatment resulted in the highest increase in roughness for G-aenial (t = 17.46, *p* < 0.001) and Estelite Super Low (t = 23.56, *p* < 0.001), suggesting that lower concentration bleaching agents can significantly alter surface texture. The one-way ANOVA confirmed these significant differences, with all materials showing *p*-values below 0.001, indicating that the type of bleaching alone strongly affects roughness. The two-way ANOVA revealed significant effects for both bleaching type (F = 87.5) and material type (F = 42.9), along with a significant interaction (F = 19.4, *p* < 0.001), meaning that the impact of bleaching on roughness varies depending on the material. Finally, the regression analysis indicated strong correlations between bleaching concentration and roughness, particularly for Estelite Medium (R^2^ = 0.92), while Estelite High showed a more moderate relationship (R^2^ = 0.69), suggesting that the effect of bleaching is highly dependent on the material’s composition.

### 3.4. Traces of Friction

The average depth of abrasion marks reveals the impact of bleaching and laser treatments on the surface integrity of dental composite materials (Table 5). G-aenial Universal Flo control samples exhibited a moderate average depth (1.16 ± 0.019), indicating a relatively resilient surface. Bleaching at 10% increased the depth to 1.25 ± 0.074, suggesting oxidative resin degradation. In contrast, 16% bleaching reduced the depth to 1.08 ± 0.065, possibly reflecting a more uniform surface response. Bleaching at 40% resulted in a slight increase to 1.18 ± 0.029, likely due to a balance between matrix breakdown and stabilization. Laser treatments generally reduced abrasion depths, with 10% + laser (1.02 ± 0.061) and 16% + laser (1.00 ± 0.029) achieving the lowest values, possibly due to enhanced cross-linking. However, 40% + laser significantly increased the depth (2.08 ± 0.048), indicating potential overheating or resin weakening.

Estelite Universal Flow High control samples exhibited a higher baseline trace depth (1.59 ± 0.077) compared to G-aenial, suggesting a softer or less wear-resistant surface. Bleaching at 10% significantly increased depth to 2.51 ± 0.091, indicating substantial matrix disruption. This effect was slightly reduced at 16% (2.18 ± 0.101), but peaked at 40% (2.68 ± 0.100), reflecting aggressive matrix decomposition. Laser treatments generally reduced trace depths, with 10% + laser (1.80 ± 0.061) and 16% + laser (1.30 ± 0.059) demonstrating substantial recovery, likely due to polymer strengthening. However, 40% + laser remained high (2.08 ± 0.085).

Estelite Universal Flow Medium control samples showed the highest initial depth (2.51 ± 0.098), indicating a rougher, potentially softer surface. Bleaching at 10% reduced depth (2.02 ± 0.064), possibly due to minor matrix restructuring. However, 16% increased depth (2.29 ± 0.105), and 40% led to the highest overall depth (2.83 ± 0.097), indicating significant surface damage. Laser treatments had varied effects, with 10% + laser (1.88 ± 0.104) reducing depth effectively, while 16% + laser (2.13 ± 0.093) and 40% + laser (2.74 ± 0.117) remained elevated, highlighting the challenge of stabilizing high-peroxide treatments.

For Estelite Universal Flow Medium, control samples had a moderate depth (1.91 ± 0.074), suggesting a balanced surface. Bleaching at 10% reduced this (1.56 ± 0.069), potentially reflecting controlled matrix reinforcement. However, 16% slightly increased depth (1.74 ± 0.044), while 40% reduced it to 1.69 ± 0.067, possibly due to filler exposure or matrix stabilization. Laser treatments consistently reduced depth, with 10% + laser (1.22 ± 0.069) achieving the lowest values, while 16% + laser (1.69 ± 0.070) and 40% + laser (1.47 ± 0.051) remained lower than their non-laser counterparts, reinforcing the potential of laser to mitigate peroxide-induced surface damage.

## 4. Discussion

One of the main observations in this study was the decrease in the microhardness of restorations after bleaching. A particularly pronounced decrease was observed in composites treated with 40% hydrogen peroxide, suggesting that high concentrations of bleaching agents may lead to degradation of the polymer matrix and weakening of the material structure. Many studies have shown a decrease in the surface hardness of composites after exposure to bleaches. Popescu et al. (2023) found significant degradation of microhardness in hybrid and nanohybrid materials after the use of CP and HP [1]. In the study of Karademir et al. (2024), bleaching using HP led to weakening of the polymer structure, reducing abrasion resistance and increasing susceptibility to microcracks [38]. Chen et al. (2024) indicated that high concentrations of hydrogen peroxide (≥35%) can cause the disintegration of polymer chains in the resin matrix, resulting in the loss of mechanical integrity of the material [39]. Similar results were obtained by Satheesh et al. (2025), who showed that bleaching both at home and in the dental office led to a significant reduction in the microhardness of composites. These researchers also emphasized that materials with a higher filler content show greater resistance to chemical degradation [40].

Other studies have shown that 10–16% bleaching solutions cause a significant decrease in microhardness, especially in composites with a low filler content [41,42]. Similar observations were also shown by Gokulnath (2025), who found that exposure to carbamide peroxide leads to degradation of the bonds between the filler and the polymer matrix, resulting in a decrease in hardness and an increase in susceptibility to microcracks [43]. In the present study, the smallest decrease was observed for Estelite Universal Flow Medium, confirming that higher filler loading increases mechanical resistance. Importantly, the microhardness of composites does not decrease proportionally to the bleach concentration; studies have shown that medium concentrations (16%) can cause greater changes than high ones (40%), which is attributed to a longer exposure time or differences in the interaction with the matrix [44,45].

The increase in surface roughness after the use of bleaching agents is a significant threat to the durability of restorations, as it may promote plaque accumulation and staining. Polydorou et al. (2006) and Abdelaziz et al. (2020) confirmed that exposure to bleaching agents results in the removal of the surface layer and the exposure of unevenness at the filler–matrix interface [46,47]. Popescu et al. (2023) reported an increase in Ra of up to 120% compared to the control sample [1], and Khan et al. (2023) showed that CP 10% and 16% caused a significantly greater effect on the surface microstructure compared to HP 35%, despite the lower concentration [48]. In this study, the highest increase in roughness was observed for Estelite Universal Flow Super Low after bleaching with 10% bleach, suggesting that long-term exposure to low concentrations may have a more destructive effect on the composite microstructure than short-term exposure to stronger preparations, in line with previous observations by Basting et al. (2007) and Attin et al. (2004), who indicated a potentially greater effect of long-term exposure to lower concentrations [49,50].

Similar observations were made by Qasim et al. (2016), Badar et al. (2024), Nunes et al. (2024), dos Santos Muniz Mota et al. (2020), and Karanasiou et al. (2021), who showed that hydrogen peroxide increases the surface roughness of composites, especially those with a low filler content [51,52,53,54,55]. Their studies also showed that composites containing nanofillers showed less structural changes after bleaching and provided greater resistance to the chemical effects of bleaches, which was confirmed in both in vitro and clinical studies, and reflected in this study—G-aenial Universal Flo, containing finer filler particles, showed a lower increase in roughness compared to Estelite Universal Flow Super Low.

Laser activation of whiteners is based on two main mechanisms: photothermal and photochemical. In the first case, the energy of laser light increases the temperature of the bleaching substance, which accelerates the decomposition of hydrogen peroxide (H_2_O_2_) into water and free oxygen radicals. These reactive molecules penetrate the structure of the composite material, oxidizing the molecules responsible for discoloration. However, excessive heating can lead to degradation of the resin matrix of the composite, causing weakening, colour change, or increased surface roughness [34,35,45]. The photochemical mechanism is based on the wavelengths of laser light, which can directly affect the dye molecules, leading to their degradation without the need to increase the temperature. Choosing the right wavelength is therefore crucial, because different composites have different absorption spectra, which can affect the effectiveness of whitening [35,55]. In practice, both mechanisms can work simultaneously, which leads to increased whitening efficiency, shorter treatment time, but also an increased risk of thermal damage in the case of improper selection of laser parameters.

There are conflicting data in the literature on the effect of laser therapy on composites; some studies suggest that the laser may initiate additional polymerization of residual monomers and strengthen the material [45,56], while others indicate that excessive heating may lead to degradation of the organic polymer matrix and enhance mechanical changes [55,57]. Montaner et al. (2024) pointed out that the increase in temperature during HP activation by laser negatively affects the stability of the polymer matrix, leading to deformation of the molecular lattice [58]. However, for composites bleached with 40% hydrogen peroxide, this effect was less pronounced, and in some cases the laser contributed to further weakening of the material structure, which is consistent with the research by Alkhudhairy et al. (2018) on the critical effect of the dose and time of laser exposure [59]. Laser therapy used in parallel with bleaching enhances degradation processes. In the study by Antunes et al. (2023), it was found that LED lasers combined with HP lead to intensified degradation of TEGDMA and Bis-GMA resin chains [60].

Tribological tests have shown that bleaching affects the abrasive properties of composites. In most cases, bleaching with 10% of the agent increased abrasion, while 16% results in the opposite. The combination of bleaching with laser gave positive effects only at low and medium concentrations (10–16%), while bleaching 40% + laser resulted in dramatic weakening of the material structure and led to significant degradation of the material, making it more susceptible to abrasion. These results are confirmed by the studies of Ertruk-Avunduk et al., (2022, 2024), Pereira et al. (2024), and Yikilgan et al. (2017), who found that laser therapy in some cases improves the mechanical resistance of composites, but its effects are strongly dependent on the type of material and the concentration of the bleach [61,62,63,64]. Also, studies conducted by Kumar et al. (2024) showed that even low concentrations of bleaches in the long term can lead to an increase in mechanical wear, especially in materials with a lower modulus of elasticity [65].

For materials such as Estelite Universal Flow High, laser therapy resulted in positive effects—Ra reduction and slower degradation. In the case of Estelite Universal Flow Super Low, the effects were the opposite—probably due to the lower thermal resistance of UDMA and TEGDMA resins without an appropriate fill factor. Kiryk et al. (2024) warn that excessive heating during laser therapy can degrade the organic resin matrix, especially in the case of high H_2_O_2_ concentrations [35]. All tested composites showed different resistances to the influence of bleaching, which confirms the importance of the material composition. Studies by Rodríguez et al. (2019) showed that nanofillers reduce the influence of chemical factors on material integrity [66]. G-aenial Universal Flo (with SiO_2_ nanoparticles and strontium glass) showed smaller changes in roughness and smaller decreases in microhardness than Estelite Universal Flow Super Low, in accordance with the studies by Moghaddam et al. (2019) [67].

From a clinical perspective, these results are of great importance. As indicated by Alrabeah et al. (2023), an increase in roughness and a decrease in surface hardness may lead to a faster loss of aesthetics of restorations and increased plaque adhesion [68]. Therefore, clinicians should be very careful when choosing bleaching methods, especially in patients with numerous composite restorations. Importantly, recent studies have shown that CP may have a detrimental effect on dental pulp stem cells, causing increased apoptosis and proliferative disorders, which is particularly important in the context of young patients [69,70,71].

Despite the interesting results, in order to maintain scientific integrity, we also point out the limitations of this study. The study was conducted in vitro, which does not fully reflect the complex conditions of the oral cavity, including changes in pH, temperature, and mechanical stresses that can significantly affect the long-term performance of dental composites. Furthermore, the study focused only on selected materials, potentially limiting the generalizability of the results to other commercially available materials with different matrix compositions, filler types, and filler contents. The study prioritized mechanical properties such as microhardness, surface roughness, and wear resistance, without addressing other critical factors such as chemical stability, colour retention, and biofilm adhesion, which are important for the clinical durability of restorative materials. Furthermore, the laser activation protocols used in this study, although effective in some cases, were not evaluated for thermal effects on the polymer matrix, which was found to lead to additional material degradation.

The findings of this study have significant clinical implications for the long-term success and durability of dental composite restorations. The observed decrease in microhardness and increase in surface roughness following exposure to bleaching agents, particularly at higher peroxide concentrations, suggest that these treatments can compromise the structural integrity and surface quality of composite materials. This degradation may lead to reduced wear resistance, increased susceptibility to microcracks, and faster loss of aesthetic properties, such as gloss and colour stability, over time. Additionally, the roughened surface can promote plaque accumulation and staining, potentially increasing the risk of secondary caries and compromising periodontal health. The partial mitigation of these negative effects by laser activation, as noted in this study, indicates that carefully controlled light-assisted bleaching may be a viable approach for minimizing composite degradation, although further research is needed to fully understand the thermal and chemical interactions involved. Clinicians should consider these factors when recommending bleaching procedures to patients with extensive composite restorations and should carefully balance aesthetic outcomes with the potential impact on material longevity.

## 5. Conclusions

The conducted studies confirm that whitening procedures significantly affect the mechanical and physicochemical properties of composite materials used in dentistry. The obtained results indicate that whitening leads to a decrease in the microhardness of composites, and the highest concentrations of hydrogen peroxide cause the greatest degradation of the material, while the composite’s resistance to whitening depends on its composition, i.e., materials with a higher content of fillers and nanoparticles show smaller changes. The increase in surface roughness after whitening may contribute to a decrease in the aesthetics and durability of fillings, and the use of a laser only partially minimizes the negative effects of whitening. In some cases, it may also contribute to further degradation of the material. Based on the obtained results, recommendations should include caution in the selection of bleaching methods and their parameters, especially in the case of using high concentrations of hydrogen peroxide in combination with a laser beam. Further studies should focus on assessing the long-term effect of bleaching on the mechanical stability of composites and the possibility of using alternative methods to improve their durability.

## Figures and Tables

**Table 1:** Material characteristics.

Name of Composite/Flowability		Type of Composite	Composite Matric	Fillers	Filler Content
Estelite Universal Flow (Tokuyama Dental, Japan)	High	Supra-Nano Spherical fillers	Bis-GMA, Bis-MPEPP, TEGDMA, UDMA	200 nm spherical SiO_2_*ZrO_2_	55 vol% 69 wt%
	Medium				57 vol% 71 wt%
	Super Low				56 vol% 70 wt%
G-aenial Universal Flo (GC, Japonia)		Microfilled	UDMA, Bis-MPEPP, TEGDMA	Silicon dioxide (16 nm) Strontium glass (200 nm)	50 vol% 69 wt%

**Table 2:** Bleaching protocol.

Type of Bleaching	Bleaching Time
Opalescence 10%	6 h × 7 days (Home bleaching)
Opalescence 16%	
Opalescence 40%	2 × 20 min + 20 min break (Office bleaching)
Opalescence 10% + laser beam	90 s of exposure (3 × 30 s + 1 min break) + 10 min after activation (Office bleaching)
Opalescence 16% + laser beam	
Opalescence 40% + laser beam	

**Table 3:** Average microhardness values before and after bleaching of filling: (a) G-aenial Universal Flo; ESTELITE UNIVERSAL FLOW (b) High, (c) Medium, (d) and Super Low.

Type of Bleaching	G-Aenial		Estelite Universal Flow High	
	Mean ± St Dev.	t-Value (*p*-Value)	Mean ± St Dev.	t-Value (*p*-Value)
Control samples	47.29 ± 0.71		42.07 ± 1.69	
bleaching 10%	45.04 ± 0.43	8.72 (<0.001)	32.32 ± 0.75	13.1 (<0.001)
bleaching 16%	43.08 ± 0.41	12.21 (<0.001)	31.18 ± 0.67	15.34 (<0.001)
bleaching 40%	33.99 ± 0.87	28.49 (<0.001)	29.68 ± 0.80	16.29 (<0.001)
bleaching 10% + laser	46.70 ± 0.44	2.47 (0.032)	38.40 ± 0.39	5.32 (<0.001)
bleaching 16% + laser	38.58 ± 1.01	9.84 (<0.001)	36.96 ± 0.44	9.92 (<0.001)
bleaching 40% + laser	38.50 ± 0.21	11.09 (<0.001)	30.70 ± 0.21	15.29 (<0.001)
One-way ANOVA	F = 106.5 *p* < 0.001		F = 251.3 *p* < 0.001	
Regression	R^2^ = 0.89		R^2^ = 0.95	
	Estelite Universal Flow Medium		Estelite Universal Flow Super Low	
	Mean ± St dev.	t-value (*p*-value)	Mean ± St dev.	t-value (*p*-value)
Control samples	45.46 ± 1.18		55.72 ± 0.94	
bleaching 10%	43.98 ± 0.79	3.02 (0.016)	46.52 ± 0.87	12.4 (<0.001)
bleaching 16%	42.56 ± 0.72	4.89 (<0.001)	45.01 ± 0.77	14.83 (<0.001)
bleaching 40%	44.37 ± 1.58	0.73 (0.479)	45.97 ± 1.29	14.38 (<0.001)
bleaching 10% + laser	45.02 ± 0.41	0.68 (0.501)	44.12 ± 2.05	11.42 (<0.001)
bleaching 16% + laser	41.46 ± 0.43	6.42 (<0.001)	46.92 ± 1.32	10.24 (<0.001)
bleaching 40% + laser	40.74 ± 0.65	8.82 (<0.001)	41.30 ± 0.38	18.66 (<0.001)
One-way ANOVA	F = 15.7 *p* < 0.001		F = 70.2 *p* < 0.001	
Regression	R^2^ = 0.75		R^2^ = 0.68	
Two-way ANOVA	F(Bleaching) = 134.8	F(Material) = 78.4	Interaction = 25.2	*p* < 0.001 (all)

**Table 4:** Averaged roughness values Ra of (a) G-aenial Universal Flo and ESTELITE UNIVERSAL FLOW (b) High, (c) Medium, and (d) Super Low restorations without bleaching and samples subjected to different bleaching procedures.

Type of Bleaching	G-Aenial		Estelite Universal Flow High	
	Mean ± St Dev.	t-Value (*p*-Value)	Mean ± St Dev.	t-Value (*p*-Value)
Control samples	0.198 ± 0.0087		0.114 ± 0.0027	
bleaching 10%	0.278 ± 0.0113	817.46 (<0.001)	0.121 ± 0.0051	3.02 (0.017)
bleaching 16%	0.209 ± 00025	4.32 (<0.001)	0.122 ± 0.0030	4.58 (<0.001)
bleaching 40%	0.205 ± 0.0131	2.81 (0.014)	0.125 ± 0.0040	6.89 (<0.001)
bleaching 10% + laser	0.227 ± 0.0072	10.49 (<0.001)	0.118 ± 0.0061	1.68 (0.136)
bleaching 16% + laser	0.216 ± 0.0051	7.81 (<0.001)	0.120 ± 0.0059	3.92 (<0.001)
bleaching 40% + laser	0.211 ± 0.0083	5.24 (<0.001)	0.117 ± 0.0025	2.38 (0.038)
One-way ANOVA	F = 68.3 *p* < 0.001		F = 12.6 *p* < 0.001	
Regression	R^2^ = 0.81		R^2^ = 0.69	
	Estelite Universal Flow Medium		Estelite Universal Flow Super Low	
	Mean ± St dev.	t-value (*p*-value)	Mean ± St dev.	t-value (*p*-value)
Control samples	0.037 ± 0.0030		0.060 ± 0.0042	
bleaching 10%	0.051 ± 0.0034	9.34 (<0.001)	0.118 ± 0.0069	23.56 (<0.001)
bleaching 16%	0.050 ± 0.0025	11.21(<0.001)	0.081 ± 0.0044	7.32 (<0.001)
bleaching 40%	0.069 ± 0.0047	18.73 (<0.001)	0.084 ± 0.0067	6.82 (<0.001)
bleaching 10% + laser	0.065 ± 0.0043	17.84 (<0.001)	0.063 ± 0.0069	0.82 (0.435)
bleaching 16% + laser	0.063 ± 0.0033	17.36 (<0.001)	0.064 ± 0.0070	0.99 (0.362)
bleaching 40% + laser	0.059 ± 0.0024	13.92 (<0.001)	0.072 ± 0.0051	2.32 (0.042)
One-way ANOVA	F = 94.7 *p* < 0.001		F = 57.8 *p* < 0.001	
Regression	R^2^ = 0.92		R^2^ = 0.78	
Two-way ANOVA	F(Bleaching) = 87.5	F(Material) = 42.9	Interaction = 19.4	*p* < 0.001 (all)

**Table 5:** Average depth of friction trace before and after bleaching: (a) G-aenial Universal Flo; ESTELITE UNIVERSAL FLOW (b) High, (c) Medium, and (d) Super Low.

Type of Bleaching	G-Aenial		Estelite Universal Flow High	
	Mean	St Dev.	Mean	St Dev.
Control samples	1.16	0.019	1.59	0.077
bleaching 10%	1.25	0.074	2.51	0.091
bleaching 16%	1.08	0.065	2.18	0.101
bleaching 40%	1.18	0.029	2.68	0.100
bleaching 10% + laser	1.02	0.061	1.80	0.061
bleaching 16% + laser	1.00	0.029	1.30	0.059
bleaching 40% + laser	2.08	0.048	2.08	0.085
	**Estelite Universal Flow Medium**		**Estelite Universal Flow Super Low**	
	Mean	St dev.	Mean	St dev.
Control samples	2.51	0.098	1.91	0.074
bleaching 10%	2.02	0.064	1.56	0.069
bleaching 16%	2.29	0.105	1.74	0.044
bleaching 40%	2.83	0.097	1.69	0.067
bleaching 10% + laser	1.88	0.104	1.22	0.069
bleaching 16% + laser	2.13	0.093	1.69	0.070
bleaching 40% + laser	2.74	0.117	1.47	0.051

## Data Availability

The original contributions presented in the study are included in the article, further inquiries can be directed to the corresponding author.
